# Supplementary material for: Assembling custom side chains on proteoglycans to interrogate their function in living cells
Source: Nat Commun. 2020 Nov 20;11:5915. doi: 10.1038/s41467-020-19765-y (PMC7679400; doi:10.1038/s41467-020-19765-y)
Supplement: Supplementary file 1 — Supplementary Information [file 41467_2020_19765_MOESM1_ESM.pdf]

Supplementary Information

Assembling Custom Side Chains on Proteoglycans to Interrogate Their Function in Living Cells

Wenshuang Wang[1], Naihan Han[1,2], Yingying Xu[1], Yunxue Zhao[3], Liran Shi[1], Jorge Filmus[4], and Fuchuan Li[1*]

Author's Affiliation

[1] National Glycoengineering Research Center and Shandong Provincial Key Laboratory of Carbohydrate Chemistry and Glycobiology, Shandong University, Qingdao, China.

[2]Shandong Police College, Jinan, China.

[3] Department of Pharmacology, School of Basic Medical Sciences, Shandong University, Jinan, China.

[4]Sunnybrook Health Science Centre, University of Toronto, Toronto, Ontario, Canada.

Correspondence

Fuchuan Li, National Glycoengineering Research Center and Shandong Provincial Key Laboratory of Carbohydrate Chemistry and Glycobiology, Shandong University, 72 Binhai Rd., Qingdao 266237, P.R. China, Tel: +86-532-58631406 Fax: +86-532-58631405, Email: fuchuanli@sdu.edu.cn.

Table of Contents

Supplementary Note 1: Confirmation of the Cys-to-fGly conversion in glypican-3 (GPC3) mutants

Supplementary Note 2: Preparation of hydrazide-labeled GAG oligosaccharides

Supplementary Note 3: Hydrazide tagging of biotin or alkyne-labeled GAG oligosaccharides

Supplementary Note 4: Optimization of reaction conditions for the assembly of GPC3 on living cell surfaces

Supplementary Note 5: Other additional data

Supplementary Note 1: Confirmation of the Cys-to-fGly conversion in GPC3 mutants

HEK293T cells were transfected with a GPC3 mutant and an hFGE expression vector (2:1 wt/wt), and the cells were lysed 2 days after transfection. After centrifugation at 15,000 × g for 10 min, the supernatant (approximately 50 μg/ml targeting proteins) was collected for further labeling of target proteins.

To probe for the presence of aldehyde, the cell lysate containing approximately 1 μg of aldehyde-tagged GPC3 mutant protein was treated with 50 mM EZ-Link™ Biotin-LC-Hydrazide (Thermo Scientific) in labeling buffer (100 μM Mes, 0.1% SDS (pH 5.5)) at room temperature for 2 h. A Corning 96-well ELISA plate was coated with avidin (0.5 μg/well) and was incubated at 4°C overnight. Then, the avidin solution was decanted, blocking buffer (3% skim milk in PBS) was added to each well and the plate was incubated for 1 h at room temperature. Next, the avidin-coated wells were incubated with biotinylated GPC3 mutants (0.3 μg) at room temperature for another 1 h. After the wells were washed three times with PBS, bound GPC3 mutants were detected using an anti-GPC3 mouse monoclonal antibody, αGCN47, followed by incubation with an HRP-conjugated goat anti-mouse IgG (Proteintech Group, Inc), and using 3,3',5,5'-tetramethyl benzidine (TMB) as a substrate.

The binding assay showed that all three mutants could efficiently bind to the avidin-coated well surface (Supplementary Figure 1), indicating that the hydrazide-reactive aldehyde group has been introduced into these mutants as expected. Furthermore, the GPC3-O mutant showed a higher avidin-binding capacity than other two mutants, probably as a result of having one more aldehyde tag.

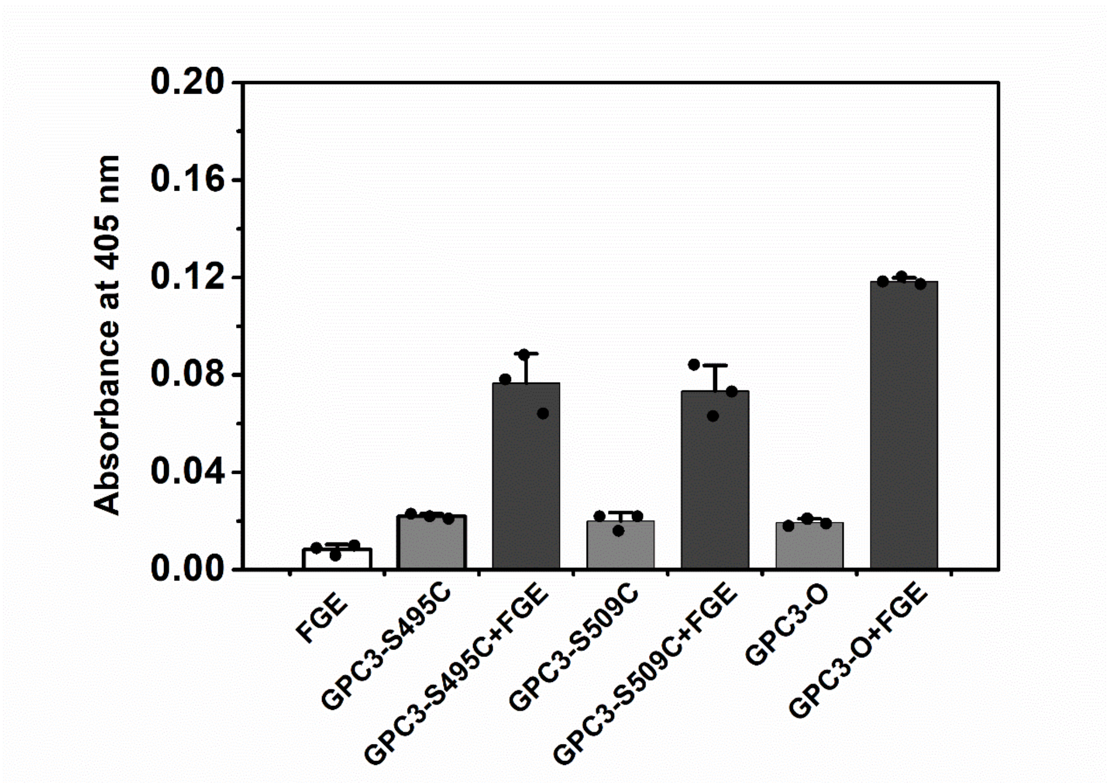

Supplementary Figure 1, Generation of aldehyde-tagged GPC3 mutants. HEK293T cells were transfected with the indicated GPC3 mutants without or with hFGE vector. Three days after transfection, the cell lysates were labeled with Biotin-LC-Hydrazide. The biotinylated GPC3 mutants were detected by a binding assay. Error bars represent means of triplicates ±S.D.

HEK293T cells were transfected with GPC3 mutants and an hFGE expression vector (2:1 wt/wt), and the cells were lysed 2 days after transfection. The supernatant was collected after centrifugation at 15,000 × g for 10 min, and then GPC3 was purified with affinity chromatography with an anti-GPC3 antibody. Briefly, aGCN (320 μg) was coupled to 200 μl of NHS-activated Sepharose (GE Healthcare) according to the manufacturer instructions. Next, a column was prepared with the coupled Sepharose. After pre-equilibration with PBS, the column was loaded with the aforementioned supernatant, washed with 50mM NaH_2PO_4-Na_2HPO_4 500mM NaCl (pH 7.2), and eluted with 0.1M triethyl amine (pH 11.5). The collected solution containing purified GPC3 mutants was neutralized by 1M NaH_2PO_4 immediately.

Purified GPC3 mutants were denatured with 6 M guanidine hydrochloride in 0.5M Tris-HCl, 2.75mM EDTA (pH 8.1), reduced with 100 mM dithiothreitol for 1 h

at 37°C, and alkylated with 150 mM iodoacetamide for 1h min at room temperature in the dark. The reaction buffer was replaced with 25 mM NH_4HCO_3 using a Microcon-10 kDa centrifugal filter. Trypsin (Promega) was added (2%, w/w) and incubated at 37 °C overnight. NanoLC-MS/MS experiments were performed on a linear ion trap–Orbitrap hybrid mass spectrometer (LTQ-Orbitrap Velos Pro, Thermo Fischer Scientific) coupled with a Nano-LC system (Shimadzu, Tokyo, Japan). Sample on-line desalting was performed using a C18 trap column (Chemicals Evaluation and Research Institute, Japan) at a flow of 50 µl/min. Reversed phase separating column (75 µm × 15 cm) was custom-made by packing 3 µm Reprosil-Pur 120 C18-AQ beads. Mobile phases consisted of 0.1% formic acid and 2% acetonitrile (A), and 0.1% formic acid and 98% acetonitrile (B). Then samples were eluted with a stepping gradient of 2 % solvent B (0.0–5.0 min); 2 to 15 % solvent B (5.0–25.0 min); 15 to 40 % solvent B (25.0–55.0 min); 40 to 98 % solvent B (55.0–60.0 min); 98 % solvent B (60.0–70.0 min); 98 to 2 % solvent B (70.0–75.0 min); and 2 % solvent B (75.0–90.0 min) at a flow of 300 nL/min.

The LTQ-Orbitrap mass spectrometer was set at a 60,000 isotopic resolution and m/z 350–1650 mass range during precursor scans. Eluted peptides from the mass spectrometer were controlled and analyzed with the Xcalibur 2.2.0 software.

As shown in Supplementary Figure 2, Cys residues underwent carbamidomethylation due to the treatment with iodoacetamide, and the doubly-charged unconverted cysteine-containing peptides N^{488}LDEEGLCTPSR^{499} and G^{500}DDEDECILCTPSR^{512}, and the FGly-containing tryptic peptides N^{488}LDEEGL(FGly)TPSR^{499} and G^{500}DDEDECIL(FGly)TPSR^{512} were identified at m/z=696.18, 834.25, 658.67 and 796.78, respectively. By analyzing the integral area of the corresponding peptide peaks by Xcalibur 2.2.0 software, the conversion efficiencies of Cys to fGly at Cys^{495} and Cys^{509} on GPC3-O were 89.2% and 87.1%, respectively. The dual conversion efficiencies of two sites in GPC3-O was 77.7% by multiplying the conversion efficiencies of Cys495 and Cys509.

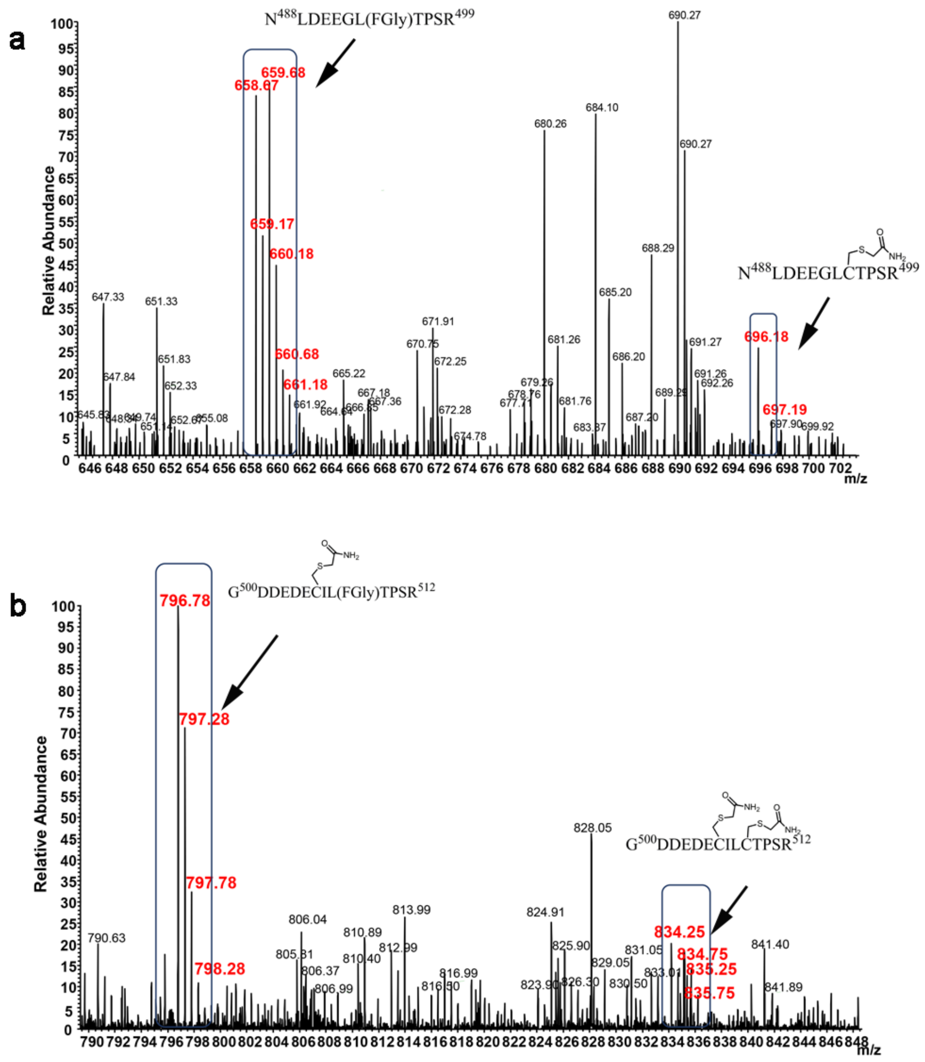

Supplementary Figure 2, Mass spectra confirming the presence of FGly in GPC3-O. **a**, Mass spectrum of the tryptic fragment N^{488}LDEEGL(FGly)TPSR^{499} incorporating FGly ([(M+2)/2], theoretical: 696.17 m/z, observed: 696.18 m/z) and its corresponding fragment N^{488}LDEEGLCTPSR^{499} incorporating unmodified Cys after treatment with 2-iodoacetamide ([(M+2)/2], theoretical: 658.67 m/z, observed: 658.67 m/z). **b**, Mass spectrum of the tryptic fragment G^{500}DDEDECIL(FGly)TPSR^{512} with carbamidomethylation incorporating FGly ([(M+2)/2], theoretical: 796.77m/z, observed: 796.78 m/z) and its corresponding fragment G^{500}DDEDECILCTPSR^{512} incorporating unmodified Cys after treatment with 2-iodoacetamide ([(M+2)/2], theoretical: 834.25m/z, observed: 834.25 m/z)

Supplementary Note 2: Preparation of hydrazide-labeled GAG oligosaccharides

In this study, various commercial GAGs were used to prepare oligosaccharides with different degrees of polymerization (DP). Their disaccharide compositions were analyzed by digestion with their corresponding lyases followed by anion-exchange HPLC as reported previously[1]. The results are shown in Table. S1**.**

To prepare the various GAG oligosaccharides, CS-A and HA (10 mg) were degraded with HCLase (5 U) in 10 ml at 30°C for 10 min[46]; DS, Hep and HS (10 mg) were degraded with chondroitinase (CSase) ABC (5 U) at 37°C, Heparinase (Hepase) I (5 U) at 30°C, and Hepase III (5 U) at 30°C for 10 min in 10 ml, respectively (Supplementary Figure 3). After the reaction, the mixture was heated in boiling water for 10 min, and subsequently cooled to 4°C. After centrifugation at 15 000×g for 30 min, the supernatant was loaded onto a pre-equilibrated Superdex 75 10/300 GL column and a Superdex Peptide 10/300 GL in tandem. Fractionated oligosaccharide samples were collected by online monitoring at 232 nm. Notably, the oligosaccharides longer than DP14 could not be effectively separated by the column, and thus were roughly separated into two fractions named Fr.1 and Fr. 2 (Supplementary Figure S4). For hydrazide group labelling at the reducing end of the oligosaccharide, each oligosaccharide fraction (20 μg) was incubated with adipic dihydrazide (200 μg) in 100 mM NaH_2PO_4-Na_2HPO_4 (pH 5.0) at 50°C for 12 h. The labeled oligosaccharides were loaded onto a NH_4HCO_3 pre-equilibrated Superdex™ Peptide 10/300 GL column, collected by online monitoring at 232 nm, and desalted by repeated freeze-drying cycles. The mix of oligosaccharides was also labeled with a hydrazide group as described above.

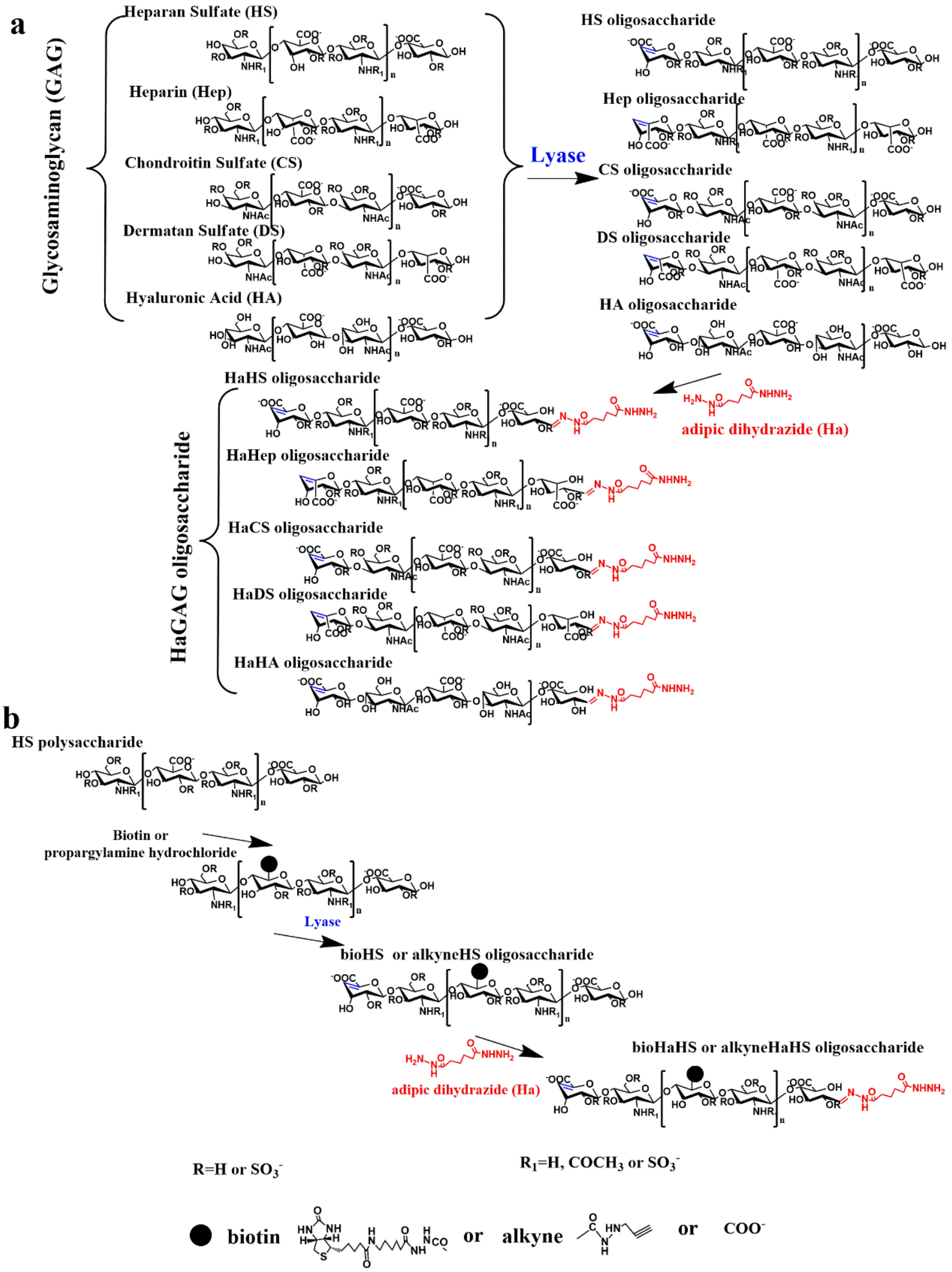

Supplementary Figure 3, Preparation and structural diagrams of various labeled oligosaccharides. a, Preparation of various hydrazide-labeled GAG oligosaccharides; **b**, Preparation of doubly labeled HS oligosaccharides.

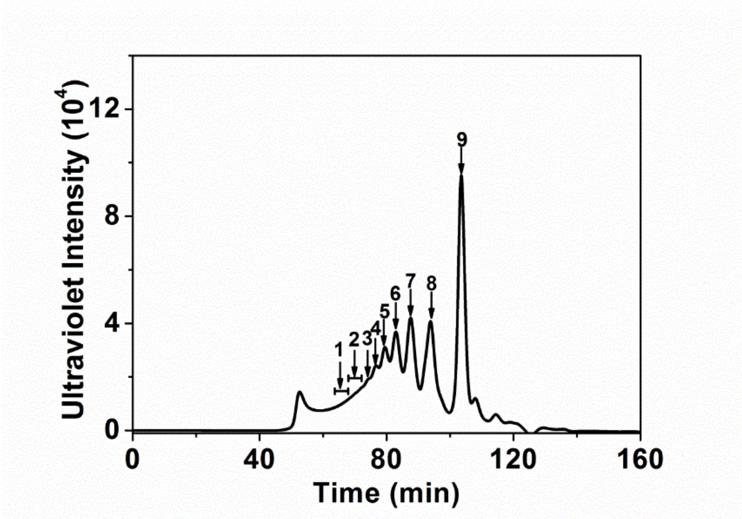

Supplementary Figure 4, Preparation of HS oligosaccharides. The HS polysaccharide was partly degraded by Hepase III, and then the products of the degradation were separated and collected by HPLC using a Superdex Peptide 10/300 GL column and a Superdex 75 10/300 GL in tandem by monitoring at 232 nm. The elution positions of the following oligosaccharides are indicated by arrows: 1, HS oligosaccharide fraction-1 (HS Fr. 1); 2, HS oligosaccharide fraction-2 (HS Fr. 2); 3, HS tetradesaccharide; 4, HS dodesaccharide; 5, HS decasaccharide; 6, HS octasaccharide; 7, HS hexasaccharide; 8, HS tetrasaccharide; 9, HS disaccharide.

In the case of small oligosaccharides such as disaccharide (DP2) and tetrasaccharide (DP4) the adipic dihydrazide-labeled products could be directly separated with gel filtration and confirmed with ESI-MS on an ion trap TOF hybrid mass spectrometer (LCMS-IT-TOF, Shimadzu, Japan). ESI-MS analysis was set in the negative ion mode with the following parameters: source voltage at 3.6 kV, nebulizer nitrogen gas flow rate at 1.5 litre/min, heat block and curve desolvation line temperature at 200°C, and detector voltage at 1.8 kV. For example, an HPLC assay showed that approximately 50% of HA disaccharides were labeled to produce a hydrazide-tagged unsaturated HA disaccharide with a corresponding m/z of 534.1933 (Supplementary Figures 5a and 5c), and about 30% of Hep disaccharides were labeled resulting in two signals-at m/z of 325.5426 and 365.5227 (Supplementary Figure 5b and 5e), which could be assigned to the hydrazide-labeled di- and trisulfated Hep disaccharides, respectively.

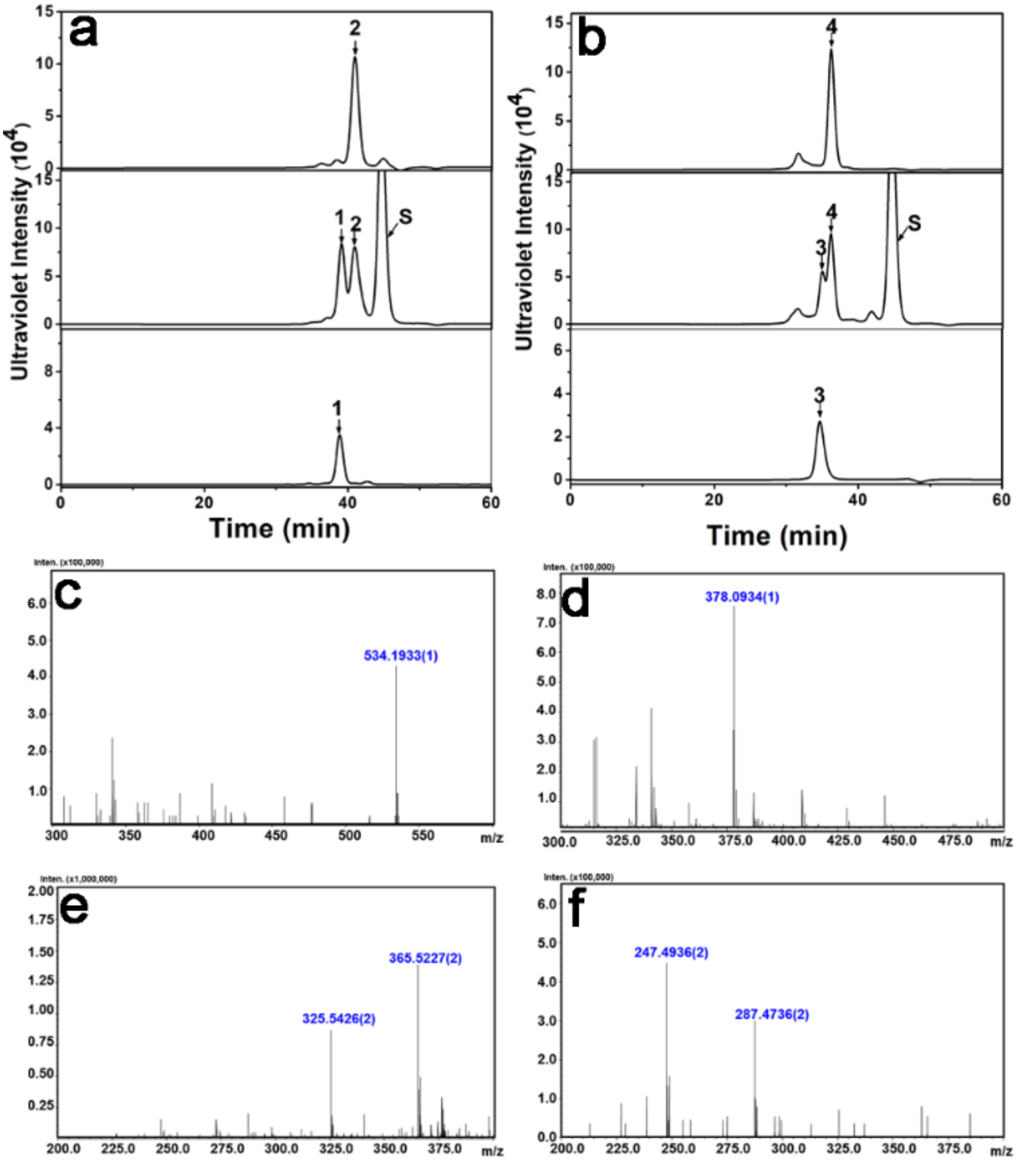

Supplementary Figure 5, Verification of the generation of GAG disaccharides labeled with an hydrazide group. a and **b,** Labeling efficiency of HA **(a)** and Hep **(b)** disaccharides without (Top panel) and with (Middle panel) adipic dihydrazide labeling was analyzed by gel filtration HPLC, and the purity of labeled disaccharides was confirmed by gel filtration HPLC (Bottom panel). **c-f,** Labeled or unlabeled disaccharides (**c**, Labeled HA disaccharide; **d**, Unlabeled HA disaccharide; **e**, Labeled Hep disaccharide; **f**, Unlabeled Hep disaccharide) were identified by ESI-MS on a LCMS-IT-TOF. 1, Hydrazide-labeled

unsaturated HA disaccharide; 2, Unsaturated HA disaccharide; 3, Hydrazide-labeled unsaturated Hep disaccharide; 4, Unsaturated Hep disaccharide; S, Salt.

In contrast, the hydrazide-labeled larger oligosaccharides cannot be efficiently separated from its corresponding unlabeled material (Supplementary Figure 6a). However, we found that the hydrazide labeling completely inhibited the cleavage of the reducing-end tetrasaccharides of GAG chains by most GAG lyases. For instance, the HS decasaccharide (20 μg) was labeled by adipic dihydrazide (200 μg) as described above, and then it was thoroughly digested by Hepase I, II and III, and the labeling efficiency of HS decasaccharides (DP10) was estimated to be about 30% as assessed by digestion with Hepases followed by a gel filtration assay (Supplementary Figure 6b), The undigested tetrasaccharide with a hydrazide tag was collected by online monitoring at 232 nm using a pre-equilibrated Superdex™ Peptide 10/300 GL column, and it was identified by ESI-MS on an ion trap TOF hybrid mass spectrometer with a negative ion mode as described above. For example, the labeled-tetrasaccharides (Supplementary Figure 6c) were identified by ESI-MS. The signals at m/z of 992.2490, 1079.1763, 1130.0625, 1173.1353, 1312.6650 correspond to hydrazide-labeled HS tetrasaccharides with two sulfate and zero acetyl groups, two sulfate and two acetyl groups, four sulfate and zero acetyl groups, four sulfate and one acetyl groups, and six sulfate and zero acetyl groups, respectively (Supplementary Figure 6d).

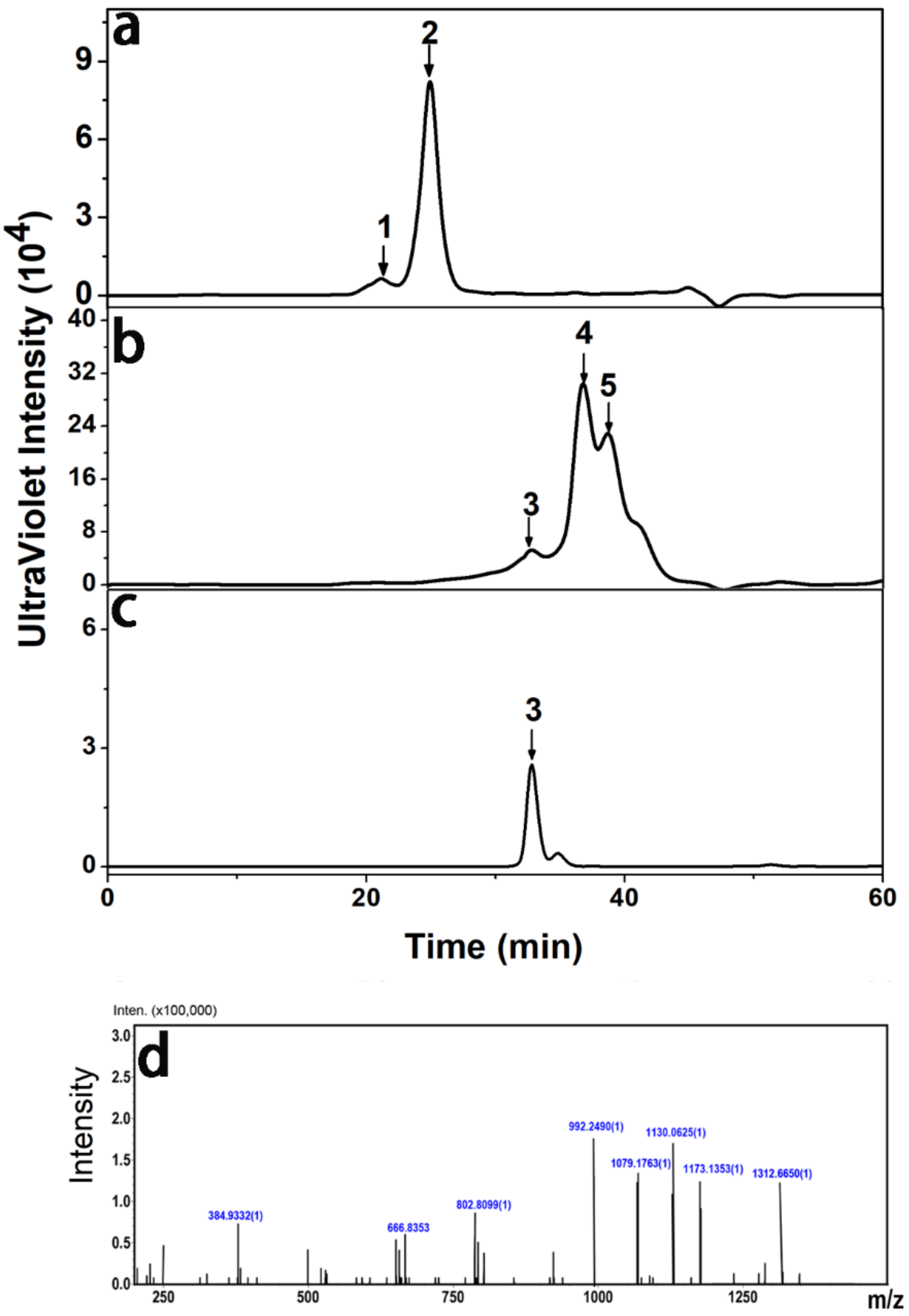

Supplementary Figure 6, Verification of the generation of HS decasaccharides labeled with an hydrazide group. a and **b, The** labeling efficiency of HS decasaccharides before (**a**) and after (**b**) digestion with Heparinase III was analyzed by gel filtration HPLC. **c**, The purity of labeled

tetrasaccharides from the reducing ends of hydrazide-labeled HS decasaccharides was confirmed by gel filtration HPLC. **d**, Labeled tetraaccharides from the reducing ends of hydrazide-labeled HS decasaccharides were identified by ESI-MS on a LCMS-IT-TOF. 1, Unsaturated HS dodecasaccharide; 2, Unsaturated HS decasaccharide; 3, Hydrazide-labeled unsaturated HS tetrasaccharide; 4, Unsaturated disulfated HS disaccharide; 5, Unsaturated monosulfated HS disaccharide.

To confirm the hydrazide-labeling of the GAG oligosaccharides, various GAG DP12 oligosaccharides were labeled by adipic dihydrazide and desalted as described above. The labelled oligosaccharide samples (1 mg) were dissolved in 0.5 ml D_2O lyophilized twice, and finally dissolved in 0.5 ml D_2O for ^1H NMR analysis. The ^1H NMR spectrum was recorded at 600 MHz on an Agilent spectrometer. Chemical shifts (δ) are given in ppm with the D_2O signal as reference. As shown in Figure. S7, the ^1H NMR spectrum of the labeled GAG oligosaccharides shows weak but significant signals at the chemical shift of 2.20-2.27 ppm corresponding to ^1H on the C2 and C5 and 1.55-1.65 ppm corresponding to ^1H on the C3 and C4 of adipic dihydrazide.

By directly analyzing the labeled products of small oligosaccharides (≤ DP4) or indirectly analyzing the labeled reducing end tetrasaccharides generated from big oligosaccharides (≥ DP6) with HPLC as described above, the labeling efficiencies of various hydrazide-labeled GAG oligosaccharides from DP2 to DP14 were determined: HA, 40.3-58.1%; CSA , 37.1-49.2%; DS, 37.8-47.3%; HS, 31.6-40.5%; and Hep, 30.2-36.9%. These results suggest that the labeling efficiency is affected by the structural features of GAGs such as sulfate degree and chain length.

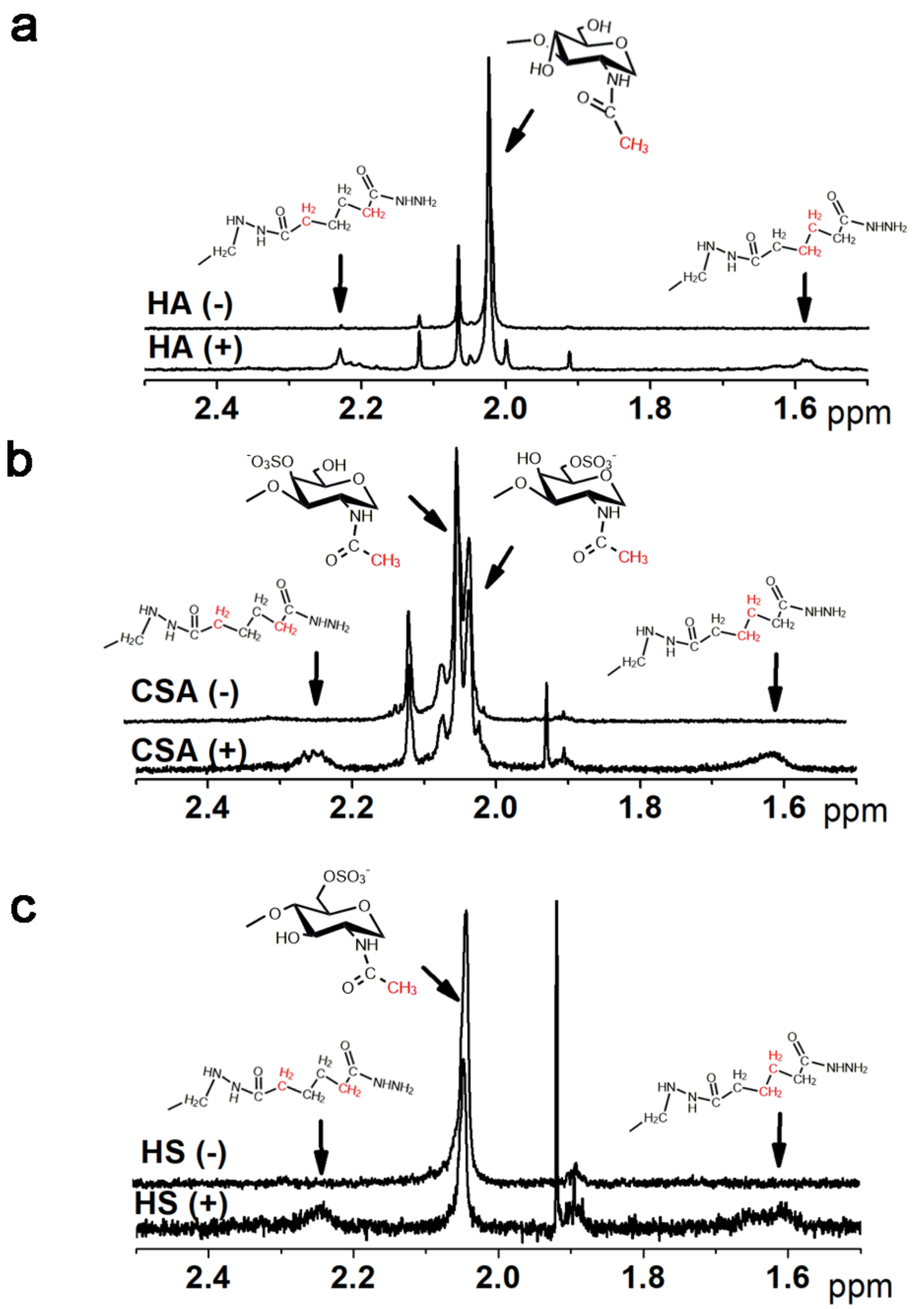

Supplementary Figure 7. ^{1}H-NMR analyses of GAG DP12 oligosaccharides labeled with hydrazide group. HA (a), CS-A (b) and HS (c) DP12 was labeled with adipic acid dihydrazide, desalted by gel filtration HPLC, and then analyzed by ^{1}H NMR spectrum.

Supplementary Table 1, Disaccharide composition of the various GAGs used in this study.

Type	Disaccharide composition					
**HA**				HexUA-GlcNAc<br>100%		
**CS-A**		HexUA-GalNAc<br>1.10%	HexUA-GalNAc(4S)<br>80.87%		HexUA-GalNAc(6S)<br>18.03%	
**DS**		HexUA-GalNAc<br>2.48%			HexUA-GalNAc(4S)<br>97.52%	
**HS**	HexUA-GlcNAc<br>11.61%	HexUA-GlcNAc(6S)<br>22.56%	HexUA-GlcNAc(NS)<br>26.55%	HexUA<br>-GlcNAc(NS,6S)<br>7.40%	HexUA<br>(2S)-GlcNAc(NS)<br>9.88%	HexUA (2S)-GlcNAc(NS,6S)<br>22.00%
**Hep**	HexUA-GlcNAc<br>3.18%	HexUA-GlcNAc(6S)<br>3.78%	HexUA-GlcNAc(NS)<br>6.56%	HexUA<br>-GlcNAc(NS,6S)<br>12.98%	HexUA<br>(2S)-GlcNAc(NS)<br>7.41%	HexUA (2S)-GlcNAc(NS,6S)<br>66.09%

HexUA: hexuronic acid; GalNAc: N-acetyl-galactosamine; GlcNAc: N-acetyl-glucosamine; 2S: 2-O-sulfate; 4S: 4-O-sulfate 6S: 6-O-sulfate NS: 2-N-sulfate.

Supplementary Note 3: Hydrazide tagging of biotin or alkyne-labeled GAG oligosaccharides

GAG polysaccharides (200 μg) were internally labelled with a biotin group using EZ-Link™ Biotin-LC-Hydrazide (final concentration 0.1 mM) according to the manufacturer's instructions (Thermo scientific). GAG polysaccharides (200 μg) were internally labelled with alkyne groups using propargylamine hydrochloride (Alfa Aesar) (final concentration 0.1 mM), EDC and NHS according to the manufacturer's instructions (Thermo Scientific). Then, the biotin- or alkyne-labeled GAG polysaccharides were moderately degraded with the corresponding enzymes, and the oligosaccharide samples were separated and collected by HPLC and desalted by repeating freeze-drying. Each oligosaccharide fraction was labelled with adipic dihydrazide as described in "Preparation of hydrazide-tagged GAG oligosaccharides" (Supplementary Figure 3).

To verify the dual labelling of HS oligosaccharides with biotin or alkyne group and hydrazide group, a Corning 96-well black ELISA plate was coated with GPC3 antibody aGCN (0.5 μg/well) and then the wells were blocked by blocking buffer (3% skim milk in PBS). Next, the wells were incubated with cell lysate containing GPC3-HaHS, GPC3-bioHaHS or GPC3-AlkyneHaHS at room temperature for 1 h. After the wells were washed three times with PBS, biotin and alkyne group labelled HS chains were detected by TRITC-streptavidin, and Cyanine3 azide through chemical click reaction, respectively. As shown in Supplementary Figure 8, TRITC-conjugated streptavidin and Cyanine3 azide could efficiently bind to the biotin- and alkyne-labeled HS oligosaccharides attached to GPC3 core proteins, respectively, indicating that HS oligosaccharides were dully labelled with hydrazide and biotin or alkyne group as expected.

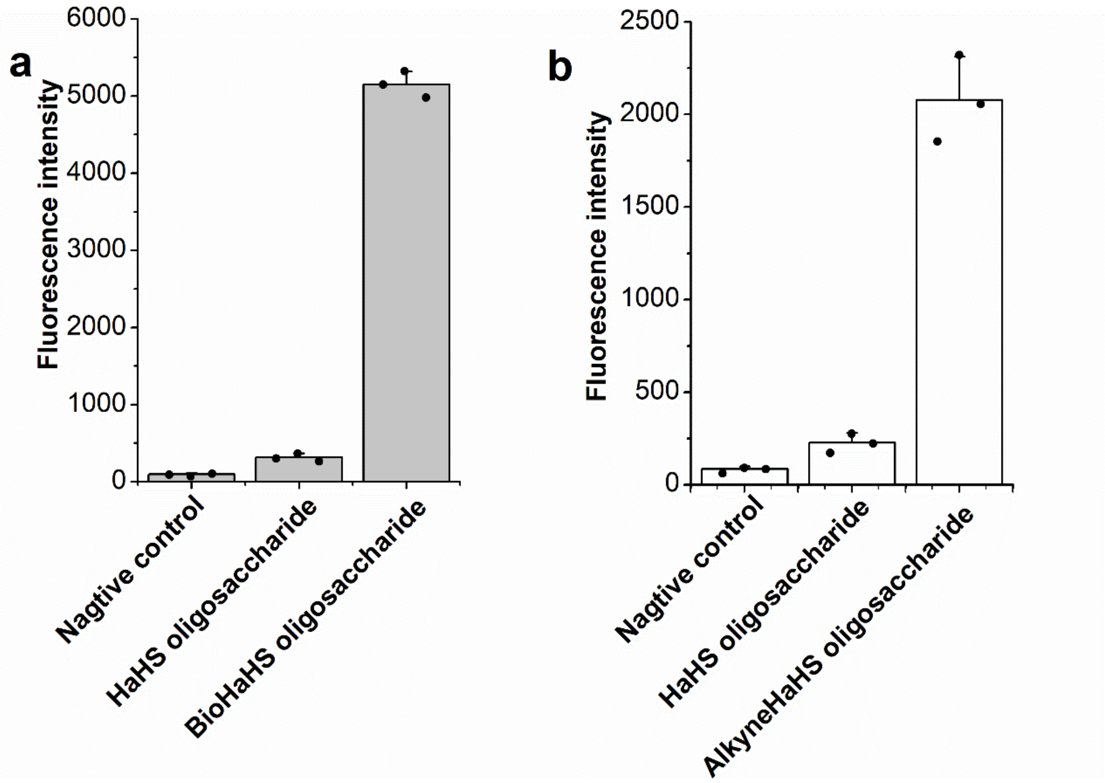

Supplementary Figure 8, Verification of the generation of doubly labeled HS chains. Cell lysates containing GPC3-HaHS, GPC3-bioHaHS or GPC3-AlkyneHaHS were added to wells coated with GPC3 antibody (aGCN), and the biotin or alkyne groups attached to the HS chains of the engineered GPC3 were detected by TRITC-streptavidin (a), or Cyanine3 azide (b) through chemical click reaction, respectively. Each experiment was done at least three times by triplicates. Error bars represent means of triplicates ±S.D.

Supplementary Note 4: Optimization of reaction conditions for the assembly of the engineered GPC3 on living cell surfaces

HEK 293T cells were seeded on 24-well plates and co-transfected with the GPC3-O or a control vector and an hFGE vector. Two days after transfection, bioHaGAG oligosaccharide chains were added to the DMEM medium (adjusted pH to 6.0) and were incubated for different times, then the medium was replaced with DMEM medium without GAG chains. The cells were suspended by gentle pipetting and were collected by centrifuging for 10 min at 1200 rpm. Cells were then blocked with blocking buffer after washing with ice-cold PBS, and were incubated with FITC-conjugated streptavidin for 1 h on ice in the dark. Finally, the cells were washed twice with PBS and analysed on a BECKMAN FC500 Flow Cytometer. The effect of conjugation conditions on the viability of cells was estimated by the Methyl tetrazolium (MTT) assay[2]. Briefly, cells were incubated in serum free DMEM medium for 4 h with 0.5 mg/ml of MTT. DMSO (150 μl) was added in the well after washing with PBS (1 ml), and then the plate was shaken gently for 10 min so that complete dissolution was achieved. Aliquots (100 μl) of the resulting solutions were transferred into 96-well plates and the absorbance was measured at 570 nm. Each experiment was done thrice by triplicates. Results are presented as percentage of the control values.

As shown in Supplementary Figure 9a, the fluorescence intensity of cells significantly increased with the oligosaccharide concetration up to 1μM and then decrease slightly, suggesting that the optimal concetration of oligosaccharide is 1μM. We also found that incubation time is a key factor for attachment efficiency. As shown in Supplementary Figure 9b, the fluorescence intensity of cells increases proportionally with the incubation time up to 4 hours. Incubation for another 4 hours only generates a modest increase in labeling. Moreover, cells' viability was 87% after treatment for 4 hours but 72% for 8 hours under the conjugation conditions (Supplementary Figure 9c). It seems, therefore, that 4 hours is the optimal time for incubation. Next, we sougth to determine the rate at which the labeled HS bound to the cell surface is lost in normal culture medium without the addition of bioHaHS

oligosaccharides. As shown in Supplementary Figure 9d, once the labeled cells were transferred into normal culture medium (pH 7.2-7.4), the fluorescence intensity gradually decreased, and after 24 hours the cells still displayed 30 % of the original fluorescence. The loss of engineered GPC3 with HS oligosaccharide chains on cell surface could be caused by the hydrolysis of the hydrazone linker, enzymatic digestion and endocytosis. The result indicates that the stability of the engineered GPC3 on the surface of cells was stable enough to allow for functional studies of GPC3 with specific GAG chains.

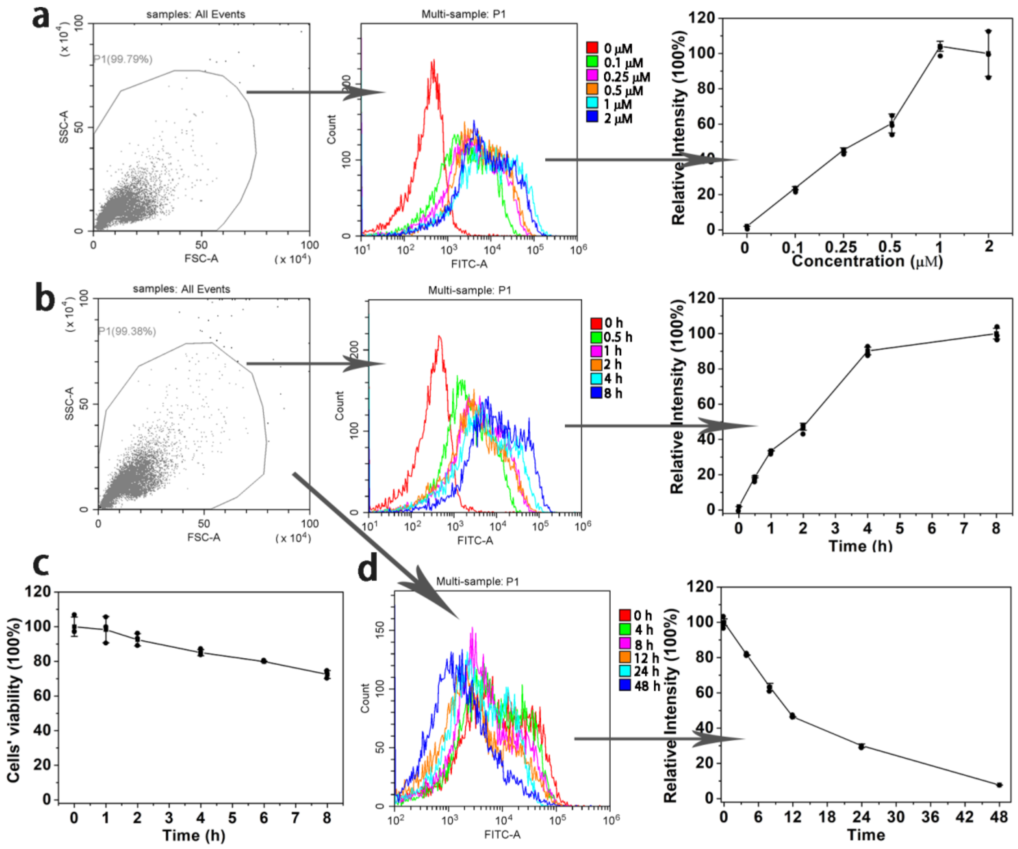

Supplementary Figure 9, Optimization of labeling conditions. GPC3-O-transfacted HEK293T cells were treated with the bioHaHS oligosaccharide mixture by using increasing concentration of oligosaccharides (a), increasing treatment time (b) and then amount of bioHaHS chains bound to the cell surface was analyzed by flow cytometry using a FITC-conjugated streptavidin probe. Each experiment was done thrice by triplicates. **c,** The effect of conjugation conditions on cells' viability. **d,** Stability of the engineered GPC3 on the cell surface. Results are shown as relative intensity compared to that of the highest intensity. Error bars represent means of triplicates ±S.D.

Supplementary Note 5: Other additional data

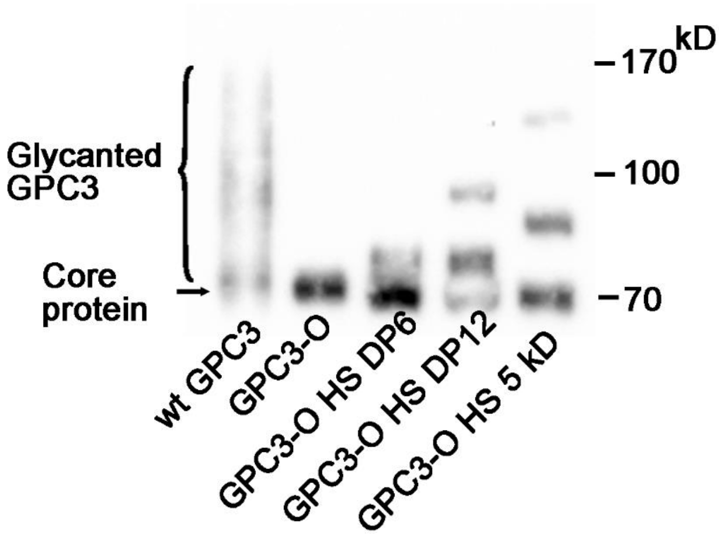

Supplementary Figure 10. Conjugation of HaHS DP6, DP12 and 5 kD (Mw) to AhGPC3-O expressed by 293T cells. The efficiency of conjugation was assessed by western blot analysis with a GPC3 antibody. wt GPC3, wild type GPC3. This experiment was repeated three times with similar results.

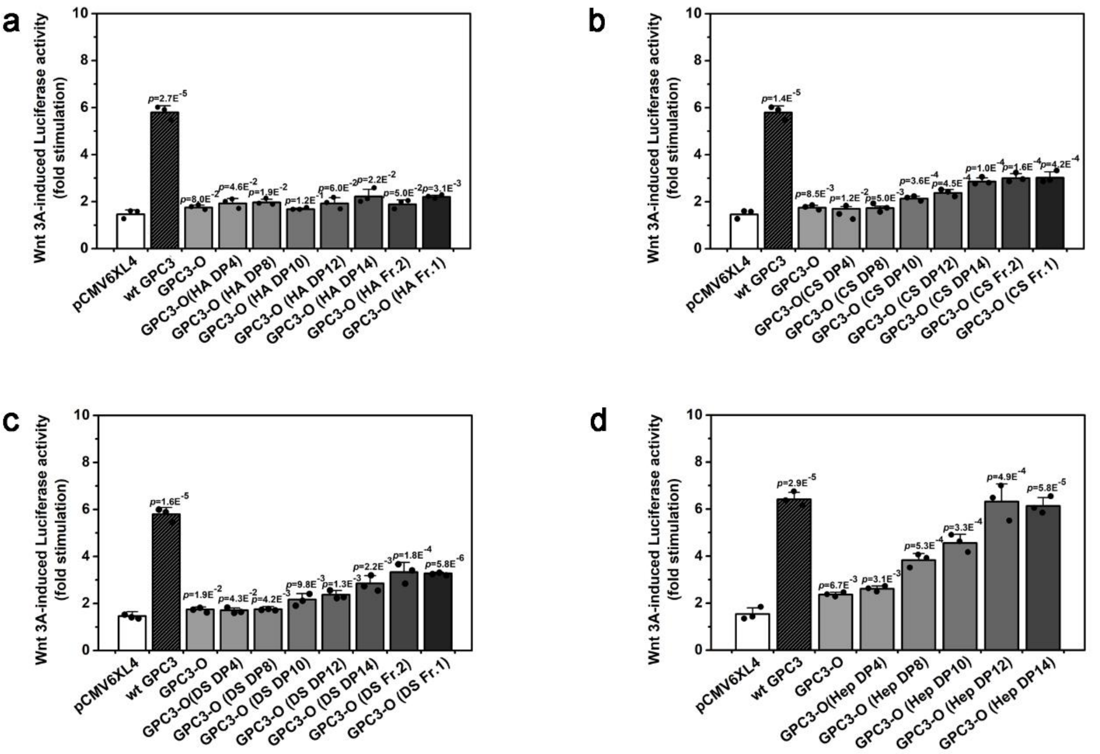

Supplementary Figure 11, Effect of various kinds of GPC3 side chains on Wnt3a cell signaling. GPC3-O-transfected HEK293T cells were treated with the indicated HaGAG oligosaccharide chains (final concentration 1μM) in culture medium (adjusted pH to 6.0) for 4 hours. Cells were then incubated for another 24 hours with control or Wnt3a CM, and the luciferase activity was measured. Each experiment was done at least three times by triplicates. Error bars represent means of triplicates ±S.D. P values in all cases are calculated by comparison to the corresponding negative control pCMV6XL4 (Two-side Student's t test). **a,** HA oligosaccharides; **b,** CS oligosaccharides; **c,** DS oligosaccharides; **d,** Hep oligosaccharides. DP4: tetrasaccharide; DP8: octasaccharide; DP10: decasaccharide; DP12: dodecsaccharide; DP14: tetradecsaccharide; Fr.2: oligosaccharide longer than DP14 but shorter than Fr.1; Fr.1: oligosaccharide longer than Fr.2. wt GPC3: wild type GPC3.

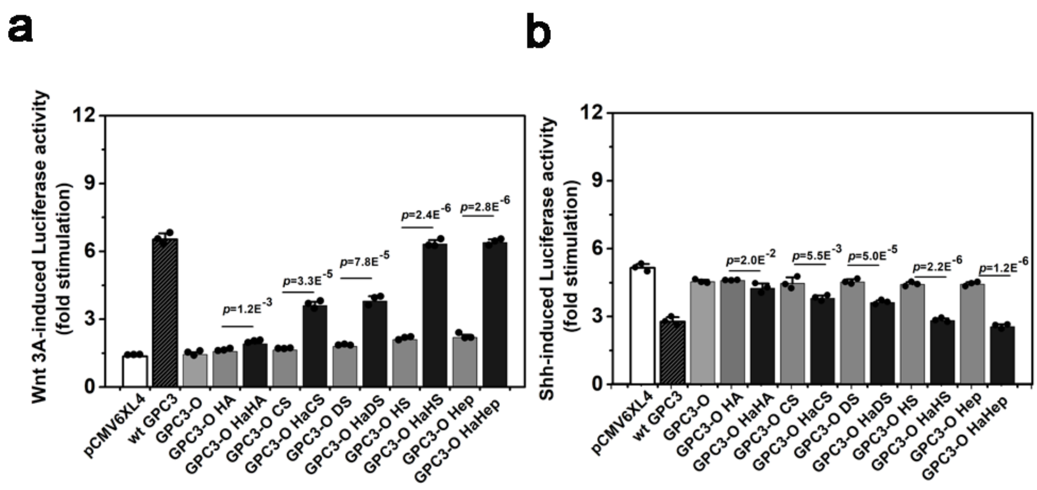

Supplementary Figure 12, Effect of different types of GAG DP14 with or without Hydrazide label on cell signaling. GPC3-O-transfected cells were treated with the indicated HaGAG or GAG DP14 oligosaccharides, and then Wnt or Shh reporter assays were performed as described above. Each experiment was done at least three times by triplicates. **a,** Wnt3A signaling pathway; **b,** Shh signaling pathway. wt GPC3: wild type GPC3. P value of HaGAG DP14 were calculated with respect to the corresponding native GAG DP14 (Two-side Student's t test). Error bars represent means of triplicates ±S.D.

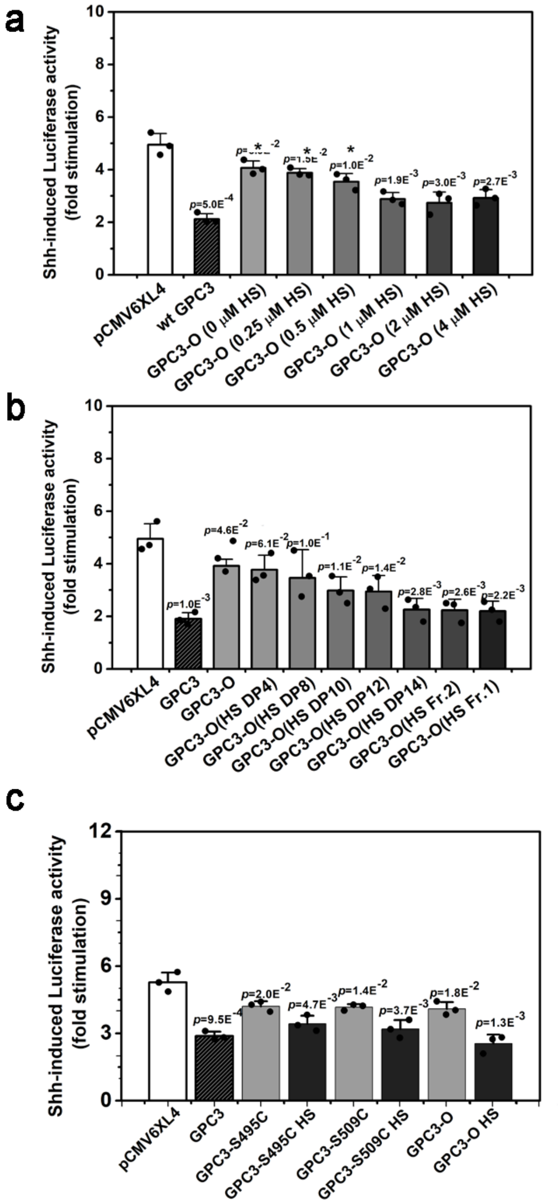

Supplementary Figure 13, Effect of GPC3 side chains on Shh cell signaling. GPC3-O-transfected NIH3T3 cells were treated with the indicated HaGAG oligosaccharide chains in culture medium (adjusted pH to 6.0) for 4 hours. Then the cells were incubated for another 24 hours with control or Shh CM, and the luciferase activity was measured. Each experiment was done at least three times by triplicates. **a,** Effect of different concentrations of HS oligosaccharide (DP14) chains; b, Effect of different Lengths of the HS oligosaccharide chains (final concentration 1μM). DP4: tetrasaccharide; DP8: octasaccharide; DP10: decasaccharide; DP12: dodecsaccharide; DP14: tetradecsaccharide; Fr.2: oligosaccharide longer than DP14 but shorter than Fr.1; Fr.1: oligosaccharide longer than Fr.2. **c,** Effect of number of HS oligosaccharide (DP14) chains. NS: not significant (p > 0.05). wt GPC3: wild type GPC3. Error bars represent means of triplicates ±S.D. P values in all case are compared with the

corresponding negative control pCMV6XL4 (Two-side Student’s t test).

Supplementary Table 2, Conjugation efficiency of HaHS oligosaccharides to AhGPC3.

**Type**	**Chain number**	**Efficiency of conjugation (%)**							
		DP2	DP4	DP6	DP8	DP10	DP12	DP14	Oligosaccharide mix
**AhGPC3-S495C**	1	-*	-	71.2	59.0	57.1	55.2	53.2	56.7
**AhGPC3-S509C**	1	-	-	72.5	58.8	57.3	55.8	54.1	57.9
**AhGPC3-O**	1	-	-	48.6	43.3	42.9	41.3	40.8	59.2
	2	-	40.8	35.1	33.3	27.7	16.9	13.2	

*Too close to the core proteins to calculate the conjugation efficiency.

Reference:

1. Kinoshita A. & Sugahara K. Microanalysis of glycosaminoglycan-derived oligosaccharides labeled with a fluorophore 2-aminobenzamide by high-performance liquid chromatography: application to disaccharide composition analysis and exosequencing of oligosaccharides. *Analytical Biochemistry* **269,** 367–378 (1999).
2. Mossmann, T. Rapid colorimetric assay for cellular growth and survival: application to proliferation and cytotoxicity assays. *Journal of Immunological Methods* **65**, 55–63 (1983).
