# Supplementary material for: Assembling custom side chains on proteoglycans to interrogate their function in living cells
Source: Nat Commun. 2020 Nov 20;11:5915. doi: 10.1038/s41467-020-19765-y (PMC7679400; doi:10.1038/s41467-020-19765-y)
Supplement: Supplementary file 3 — Reporting Summary [file 41467_2020_19765_MOESM3_ESM.pdf]

nature research

Corresponding author(s): Fuchuan Li

Last updated by author(s): Oct 20, 2020

Reporting Summary

Nature Research wishes to improve the reproducibility of the work that we publish. This form provides structure for consistency and transparency in reporting. For further information on Nature Research policies, see Authors & Referees and the Editorial Policy Checklist.

Statistics

For all statistical analyses, confirm that the following items are present in the figure legend, table legend, main text, or Methods section.

n/a	Confirmed	
☐	☒	The exact sample size (*n*) for each experimental group/condition, given as a discrete number and unit of measurement
☐	☒	A statement on whether measurements were taken from distinct samples or whether the same sample was measured repeatedly
☐	☒	The statistical test(s) used AND whether they are one- or two-sided<br>*Only common tests should be described solely by name; describe more complex techniques in the Methods section.*
☒	☐	A description of all covariates tested
☒	☐	A description of any assumptions or corrections, such as tests of normality and adjustment for multiple comparisons
☒	☐	A full description of the statistical parameters including central tendency (e.g. means) or other basic estimates (e.g. regression coefficient) AND variation (e.g. standard deviation) or associated estimates of uncertainty (e.g. confidence intervals)
☐	☒	For null hypothesis testing, the test statistic (e.g. *F*, *t*, *r*) with confidence intervals, effect sizes, degrees of freedom and *P* value noted<br>*Give P values as exact values whenever suitable.*
☒	☐	For Bayesian analysis, information on the choice of priors and Markov chain Monte Carlo settings
☒	☐	For hierarchical and complex designs, identification of the appropriate level for tests and full reporting of outcomes
☒	☐	Estimates of effect sizes (e.g. Cohen's *d*, Pearson's *r*), indicating how they were calculated

Our web collection on statistics for biologists contains articles on many of the points above.

Software and code

Policy information about availability of computer code

Data collection	Zeiss Zen software (2012 S4)-confocal image acquisition<br>CytExpert (Beckman CoulterCytoFLEX)-flow cytometry acquisition<br>Labsolutions (Shimadzu)-HPLC data acquisition<br>FCQ (CELL biosciences)-western blot acquisition<br>Agilent 600MHz-NMR data acquistion<br>Xcalibur (2.2.0)-NanoLC-MS/MS data acquistion
Data analysis	Zeiss Zen software (2012 S4)-confocal image preparation<br>Excel 2019 (Microsoft), Origin 8.5 (OriginLab)-figure preparation and statistical test calculation<br>AlphaView Q (CELL biosciences)-western blot analysis<br>FlowJo 10-flow cytometry analysis<br>LC solution version1.25-HPLC data acquisition<br>image J (v1.8.0)-image analysis<br>Xcalibur (2.2.0)- NanoLC-MS/MS data analysis<br>MestReNova (9.0.1)-NMR data analysis<br>ChemBioDraw Ultra 12.0- generation of structural diagrams of various labeled oligosaccharides<br>Adobe Photoshop 13.0-figure preparation

For manuscripts utilizing custom algorithms or software that are central to the research but not yet described in published literature, software must be made available to editors/reviewers. We strongly encourage code deposition in a community repository (e.g. GitHub). See the Nature Research guidelines for submitting code & software for further information.

Data

Policy information about availability of data

All manuscripts must include a data availability statement. This statement should provide the following information, where applicable:

- Accession codes, unique identifiers, or web links for publicly available datasets
- A list of figures that have associated raw data
- A description of any restrictions on data availability

The data that support the findings of this study are available from the corresponding author upon reasonable request.

Field-specific reporting

Please select the one below that is the best fit for your research. If you are not sure, read the appropriate sections before making your selection.

☒ Life sciences ☐ Behavioural & social sciences ☐ Ecological, evolutionary & environmental sciences

For a reference copy of the document with all sections, see nature.com/documents/nr-reporting-summary-flat.pdf

Life sciences study design

All studies must disclose on these points even when the disclosure is negative.

Sample size	Sample size was determined based on similar studies in this field.
Data exclusions	Data points were excluded when there was a technical mistake during the experimental procedure.
Replication	Experiments were performed in at least three independent biological replicates.<br>All attempts at replication were successful and gave similar results.
Randomization	Randomization was not used and relevant to this study, since it did not involved an allocation of an intervention or a trial with human or animal participants.
Blinding	Blinding was not used and relevant to this study, since it did not involved an allocation of an intervention or a trial with human or animal participants

Reporting for specific materials, systems and methods

We require information from authors about some types of materials, experimental systems and methods used in many studies. Here, indicate whether each material, system or method listed is relevant to your study. If you are not sure if a list item applies to your research, read the appropriate section before selecting a response.

Materials & experimental systems

n/a	Involved in the study	
☐	☒	Antibodies
☐	☒	Eukaryotic cell lines
☒	☐	Palaeontology
☒	☐	Animals and other organisms
☒	☐	Human research participants
☒	☐	Clinical data

Methods

n/a	Involved in the study	
☒	☐	ChIP-seq
☐	☒	Flow cytometry
☒	☐	MRI-based neuroimaging

Antibodies

Antibodies used	An anti-GPC3 mouse monoclonal antibody (αGCN) was prepared in our laboratory (Han et.al Chem Commun. 2017). An HRP-conjugated goat anti-mouse (IgG) secondary antibody (Cat#:SA00001-1, Lot# 20000002) and an anti-Frizzled-7 rabbit antibody (Cat#: 16974-1-AP, Lot#: 00019958) were purchased from Proteintech Group (Rosemont, USA). An anti-Wnt3a rabbit antibody (Cat#:ab28472, Lot#: GR3187814-1), an anti-Shh rabbit antibody (Cat#: ab53281, Lot#: GR3188172-1), an Alexa Flour 405-conjugated goat anti-rabbit (IgG) secondary antibody (Cat#: ab175652; Lot#: GR319304-3), and a Alexa Flour 488-conjugated goat anti-mouse (IgG) secondary antibody (Cat#:ab150113, Lot#: GR315193-1) were obtained from Abcam (Shanghai, China)
Validation	Previous validations and citations can be found by using the RRID numbers. An HRP-conjugated goat anti-mouse (IgG) secondary antibody (RRID:AB_2722565) and an anti-Frizzled-7 rabbit antibody (RRID:AB_2294535), An anti-Wnt3a rabbit antibody (RRID:AB_2215308), an anti-Shh rabbit antibody (RRID:AB_882648), an Alexa Flour 405-conjugated goat anti-rabbit (IgG) secondary antibody (RRID:AB_2687498), and a Alexa Flour 488-conjugated goat anti-mouse (IgG) secondary antibody

(RRID:AB_2576208), . An anti-GPC3 mouse monoclonal antibody (αGCN) was prepared in our laboratory (Han et.al Chem Commun. 2017).

Eukaryotic cell lines

Policy information about cell lines

Cell line source(s)	L and L Wnt3A from mouse subcutaneous connective tissue; 293T cell from human kidney; NIH3T3 cell from mouse embryo; 293T cell was obtained from Cell bank of typical culture preservation committee, Chinese academy of sciences; L, L Wnt3A and NIH3T3 were obtained from ATCC
Authentication	Authentication was not performed for this study.
Mycoplasma contamination	Negative for mycoplasma contamination
Commonly misidentified lines (See ICLAC register)	No commonly misidentified cell lines were used

Flow Cytometry

Plots

Confirm that:

- [x] The axis labels state the marker and fluorochrome used (e.g. CD4-FITC).
- [x] The axis scales are clearly visible. Include numbers along axes only for bottom left plot of group (a 'group' is an analysis of identical markers).
- [x] All plots are contour plots with outliers or pseudocolor plots.
- [x] A numerical value for number of cells or percentage (with statistics) is provided.

Methodology

Sample preparation	The cells were suspended by gentle pipetting; then, they were collected by centrifuging for 5 min at 1200 rpm, blocked with blocking buffer after washing with ice-cold PBS, and then were incubated with FITC-conjugated streptavidin for 1 h at room temperature in the dark. Finally, the cells were washed twice with PBS and analysed.
Instrument	Beckman CoulterCytoFLEX
Software	CytExpert
Cell population abundance	The total collected and analyzed cell numbers were 10000 per sample. This number was according the FSC and SSC gate after removing the dead and clumped cells.
Gating strategy	First, gate the FSC and SSC as P1 gate after removing the dead and clumped cells. Second, analyzed all cells fluorescence intensity in P1 gate.

- [ ] Tick this box to confirm that a figure exemplifying the gating strategy is provided in the Supplementary Information.
